# A case series of distal renal tubular acidosis, Southeast Asian ovalocytosis and metabolic bone disease

**DOI:** 10.1186/s12882-020-01959-7

**Published:** 2020-08-05

**Authors:** WMSN Gunaratne, DMDIB Dissanayake, KADS Jayaratne, NP Premawardhana, Sisira Siribaddana

**Affiliations:** 1 Professorial Medical Unit Teaching Hospital Anuradhapura, Anuradhapura, Sri Lanka; 2 Medical unit A Teaching Hospital Anuradhapura, Anuradhapura, Sri Lanka; 3 Consultant hematologist Teaching Hospital Anuradhapura, Anuradhapura, Sri Lanka; 4 Faculty of Medicine and Allied Sciences, Rajarata University of Sri Lanka, Anuradhapura, Sri Lanka

**Keywords:** Distal renal tubular acidosis, Southeast Asian ovalocytosis, Hypokalemic paralysis, Normal anion gap metabolic acidosis, Delta ratio, Chronic kidney disease, Medullary nephrocalcinosis, Metabolic bone disease, Osteosclerosis, Case reports

## Abstract

**Background:**

Familial distal renal tubular acidosis (dRTA) associated with mutations of solute carrier family 4 membrane − 1 (*SLC4A1*) gene could co-exist with red cell membrane abnormality, Southeast Asian ovalocytosis (SAO). Although this association is well described in Southeast Asian countries, it is less frequently found in Sri Lanka.

**Case presentation:**

We describe six patients who had dRTA co-existing with SAO. All of them initially presented with severe hypokalemia and paralysis. They presented within a period of six months to the Teaching Hospital Anuradhapura, Sri Lanka.

All had metabolic acidosis indicated by low serum bicarbonate. Three of them were having underlying chronic kidney disease as well. Those three patients had mixed high and normal anion gap metabolic acidosis indicated by low delta ratio. In all dRTA was confirmed by presence of normal anion gap, hyperchloraemia, high urine pH and positive urine anion gap.

Examination of blood films of all of them revealed presence of stomatocytes and macro-ovalocytosis compatible with SAO.

In relation to complications of dRTA, two patients had medullary nephrocalcinosis. Three patients had biochemical evidence of osteomalacia, with two of them having radiological evidence of diffuse osteosclerosis. One patient had secondary hyperparathyroidism and a pathological fracture.

**Conclusions:**

Erythrocyte in SAO is exceptionally rigid and this abnormality is said to be evolved as it protects against *Plasmodium vivax* malaria and cerebral malaria cause by *Plasmodium falciparum*. Although two families of SAO was described earlier, SAO and dRTA combination was reported only once in a patient from Anuradhapura district.

Distal renal tubular acidosis, SAO combination and its related complications including nephrocalcinosis, chronic kidney disease and metabolic bone disease was not described in Sri-Lankan literature. This case series emphasize the importance of investigating recurrent/ chronic hypokalemia to diagnose dRTA and its associations, as early correction of acidosis could prevent development of chronic kidney disease and metabolic bone disease.

## Background

Distal renal tubular acidosis (dRTA) is characterized by impaired hydrogen ion secretion in distal nephron. This defect leads to an inability to excrete acid load causing hydrogen ion retention and hyperchloremic metabolic acidosis with an inappropriately alkaline urine. Distal RTA is commonly associated with hypokalemia causing muscle paralysis which could be the first manifestation of the underlying disease.

Mutations in genes encoding renal acid-base transporters have been identified as causes of inherited/primary dRTA [1]. These secretory defects may be observed with autosomal dominant or recessive transmissions. Autosomal dominant dRTA has been found to be associated with mutations in the gene encoding the Cl−−HCO3− exchanger *AE1* (*SLC4A1* gene product) or band 3 protein. In the kidney the protein is involved in acidification of the urine and in the red cell it functions both as an anion exchanger and as a membrane glycoprotein required for shape and stability. Autosomal recessive dRTA which may be associated with sensorineural deafness present mutations in the gene *ATP6B1* encoding the B1-subunit of H + -ATPase. Autosomal recessive dRTA without sensorineural deafness may be caused by mutations in the gene *ATP6N1B* encoding the 116-kD subunit of H+ ATP-ase. Recent genetic studies has shown that autosomal recessive dRTA associated with *AE1* gene mutation in many countries in Southeast Asia. Accordingly both dominant and recessive forms of dRTA can be associated with mutation of the *SLC4A1* gene [2]. Several mutations of the red-cell anion exchanger band 3 (*AE1*, *SLC4A*1) gene associated with dRTA are reported worldwide. Three mutations, (G701D), (A858D) and deletion of Val^850^ (DV850) described are restricted to South-East Asian populations particularly to Malaysia and Papua New Guinea [3].

Familial dRTA and an inherited erythrocyte disorder ‘Southeast Asian Ovalocytosis’ (SAO), both caused by mutations in the solute carrier family 4 membrane − 1 (*SLC4A1*) gene, may be co-inherited in trans resulting in dRTA [2]. SAO is an inherited erythrocyte disorder characterized by macro-ovalocytes and stomatocytes on blood smear. It results from the band 3 deletion of residues 400–408 (B3 SAO) which is responsible for an unusual erythrocyte stiffness and oval shape of the cells on blood smear. Heterozygous carriers are usually asymptomatic whereas homozygous are usually lethal and appear to be incompatible with life.

Mutations in *SLC4A1* gene associated with dominant dRTA do not affect the function of the erythroid isoform band 3 unless the patient has an additional deletion causing SAO. Out of three gene mutations of band 3 mentioned above, G701D and DV850 have autosomal recessive inheritance but segregate as though dominant when the B3 SAO allele is present which is called Pseudo-dominant phenotype [3]. A858D mutation has autosomal dominant inheritance. Patients with dRTA may be homozygous for the mutation or be compound heterozygotes of two different *AE1* mutations. Compound heterozygotes with B3 SAO exhibit dRTA co-existing with SAO. Mutations exert different effects on the biosynthesis, structure and function of band 3 that lead to defective acid secretion in the kidney [4].

We report six patients presenting with hypokalemic paralysis, dRTA and SAO. All patients are from Anuradhapura district in the North Central Province of Sri Lanka, and presented to the Teaching Hospital Anuradhapura within a period of 6 months. Four of them had notable past medical history of hypokalemic periodic paralysis but previously the presence of SAO was not evaluated. Their acid base disturbances were re-evaluated. All belong to ethnic group Sinhala. None of them had evidence of an underlying autoimmune disease or hearing impairment. Except for the 4th patient who had family history of undiagnosed renal disease, none of the patients had family history of similar illness or consanguinity.

## Case presentation

### Patient 1

**Fig. 1:**
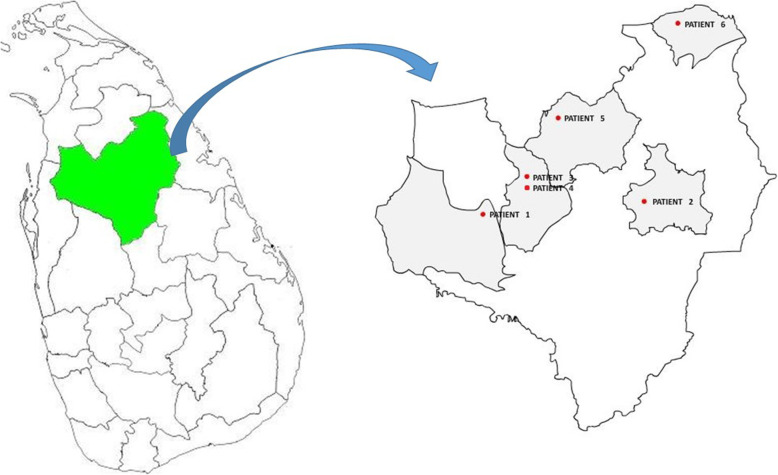
Map of Sri Lanka and Anuradhapura district showing residential areas of 6 patients. (created using Adobe Photoshop CS5)

A 49-year-old female from Nochchiyagama (Fig. 1) was admitted with progressive weakness of all four limbs for 3 days. This was her fourth admission with paralysis of limbs during last 2 years. Her past medical records revealed recurrent admissions with hypokalemia and evidence of distal renal tubular acidosis (dRTA) at her initial presentation in 1995 (Table 1). She was on potassium chloride tablets and potassium citrate solution with very poor compliance. In 2014 she developed a right upper femoral fracture following a minor trauma, which was corrected by a dynamic hip screw.

**Table 1:** Investigations of the six patients (abnormal values are in bold)^a^

Investigations	Patient 1		Patient 2	Patient 3	Patient4	Patient 5	Patient 6
Year presented	1995	2016	2016	2016	2016	2016	2016
K^+^ mmol/L (3.5–5)	**1.5**	**2.7**	**2.3**	**2.1**	**2.5**	**2**	**2.4**
Na^+^mmol/L (135–145)	135	138	135	140	140	136	140
Cl^−^ mmol/L (96–106)	**110**	**109**	**110**	106	**108**	**110**	**112**
pH (7.35–7.45)	**7.30**	**7.28**	7.40	7.40	**7.30**	7.42	7.40
pCO_2_ mmHg (35–45)	**26**	**20**	**30**	**28**	**30**	**31**	**32**
HCO_3_ mmol/L (20–28)	**13.0**	**8.8**	**16.0**	**14.6**	**15.7**	**16.0**	**16.5**
Anion gap^1^ (4–12)	12.0	**20.2**	9.0	**19.4**	**16.3**	10.4	11.5
Delta ratio^2^	0	0.53	−1.1	0.78	0.5	−0.2	−0.06
Urine pH^3^ (4.5–8)	6.5	6.5	6.8	6.5	6.6	7.5	7.5
Urine K^+^ mmol/L(25–125)	32	40	75	55	45	46	30
Urine Na^+^ mmol/L(50–125)	54	102	111	85	130	90	110
Urine Cl^−^ mmol/L(25–40)	**55**	**85**	**110**	**105**	**120**	**58**	**65**
UAG^4^	31	57	76	35	55	78	75
Serum Creatinine μmol/L (eGFR ml/min)	88 (70)	**126 (39)**	60 (89)	**236 (25)**	**125 (58)**	94 (72)	86 (61)
Ultra sound scan abdomen	Normal	**B/L MN**^**b**^ **CKD**	Normal	**CKD**	**CKD**	**B/L MNb**	Normal
Ca^++^ mmol/L (2.1–2.6)	2.12	2.10	2.10	**1.94**	**1.95**	2.50	2.10
PO_4_ mmol/l (0.8–1.5)	**0.6**	1.13	0.8	0.94	**0.5**	1.2	1.2
ALP u/l (80–380)	84	**1958**	86	**550**	**807**	103	**544**
TSH (0.4–4) micro Iu/ml	NA	Normal	Normal	Normal	Normal	Normal	**6.31**
PTH pg/ml (8.8–76.6)	NA	**96.8**	24.7	**78**	28.7	NA	NA
X-ray		**Reduced bone density, loosers zones and pathological fracture**	Normal	**Diffuse osteo-sclerosis**	**Diffuse osteo-sclerosis**	Normal	Normal
Haemoglobin (g/dL)		**9.3**	11.3	**10.6**	13	13.6	12.4
Mean corpuscular volume (MCV) (80-96 fl)		**106.6**	**103**	**109**	**103.9**	**105**	**98**
Reticulocyte count (0.5–1.5%)		1.5	1.2	1.4	1.2	0.9	1.2
Blood Film		**SAO**	**SAO**	**SAO**	**SAO**	**SAO**	**SAO**

^1^Anion gap = Na - (Cl + HCO3)

^2^Delta Ratio = change in Anion Gap (AG-12)/change in bicarbonate (24-[HCO_3_^−^]) [5]

Interpretation

< 0.4 = hyperchloraemic normal anion gap metabolic acidosis

0.4–0.8 = combined normal & high anion gap metabolic acidosis

1–2 = uncomplicated high anion gap metabolic acidosis

> 2 = could be due to a pre-existing metabolic alkalosis, or to compensation for a pre-existing respiratory acidosis (ie compensated chronic respiratory acidosis)

^3^Depending on the person’s acid-base status, the pH of urine may range from 4.5 to 8. Patients with normal renal function and normal renal acidification mechanisms who develop metabolic acidosis usually have a urine pH of 5.3 or less

^4^Urine anion gap (UAG) = Urine (Na + K-Cl). A positive UAG is consistent with low or normal NH^4^ excretion and a negative UAG is consistent with increased NH^4^ excretion

^a^Urine microscopy was normal and sediment was bland in all patients. Serum albumin was within normal limits in all patients

^b^*B/L MN* Bilateral medullary nephrocalcinosis

On examination she had flaccid paralysis of all four limbs but her tendon reflexes, plantar responses and sensory examination was normal. Remainder of physical examination was unremarkable except for mild pallor. Her serum potassium value on admission was 2.7 mmol/l and her symptoms improved with potassium replacement.

**Fig. 2:**
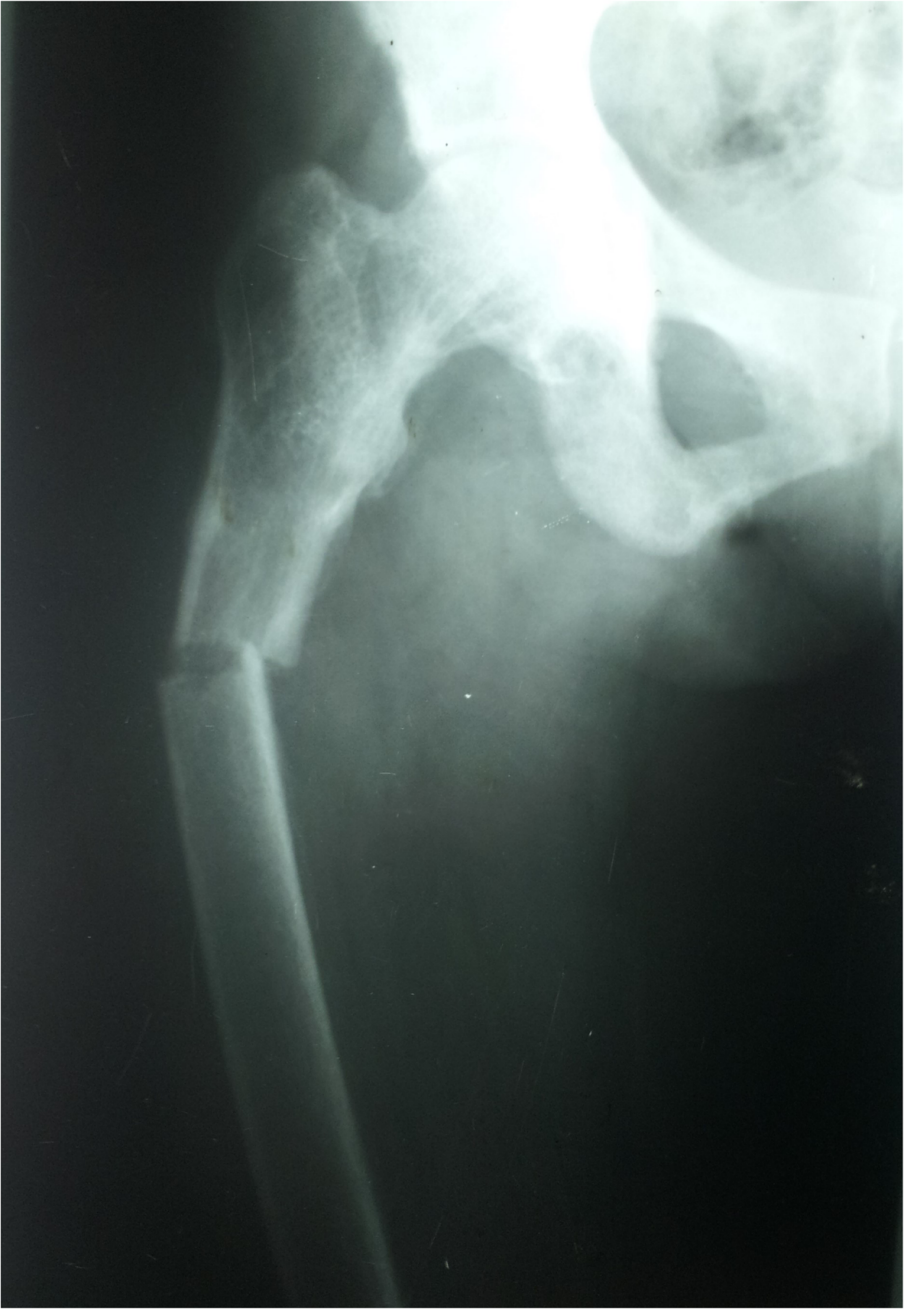
Right femoral pathological fracture

**Fig. 3:**
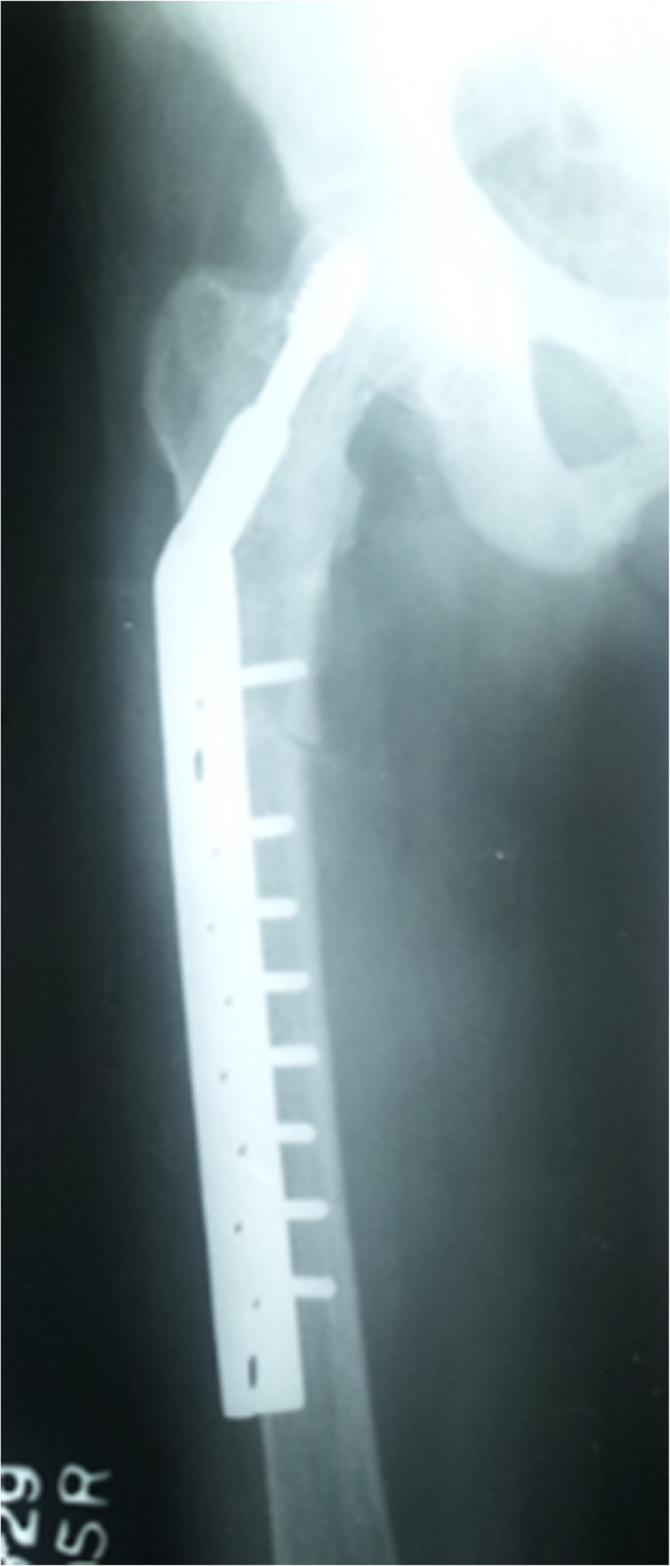
Fracture corrected with DHS

**Fig. 4:**
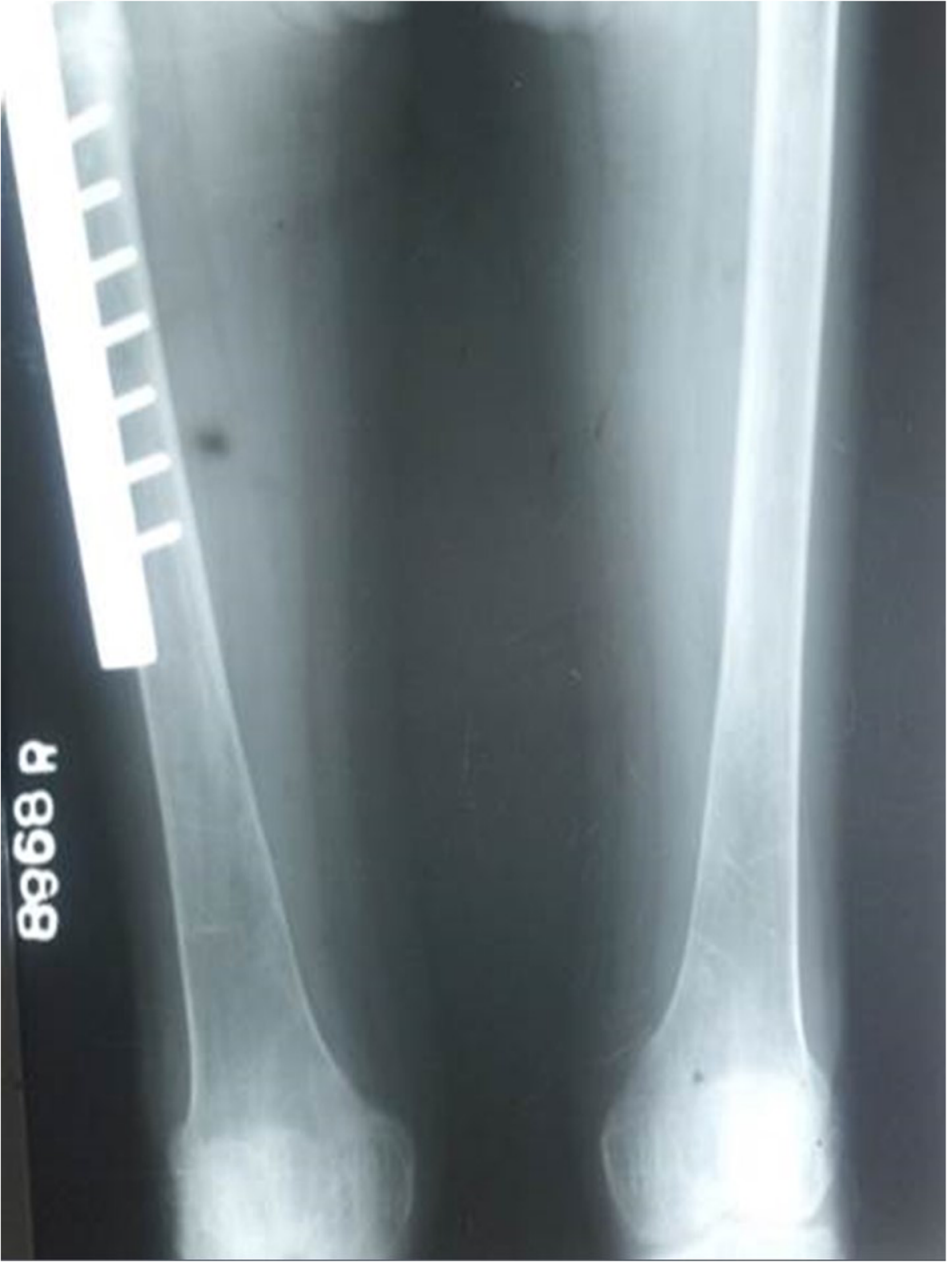
reduced bone density, loosers zones, and pathological fracture compatible with osteomalacia

**Fig. 5:**
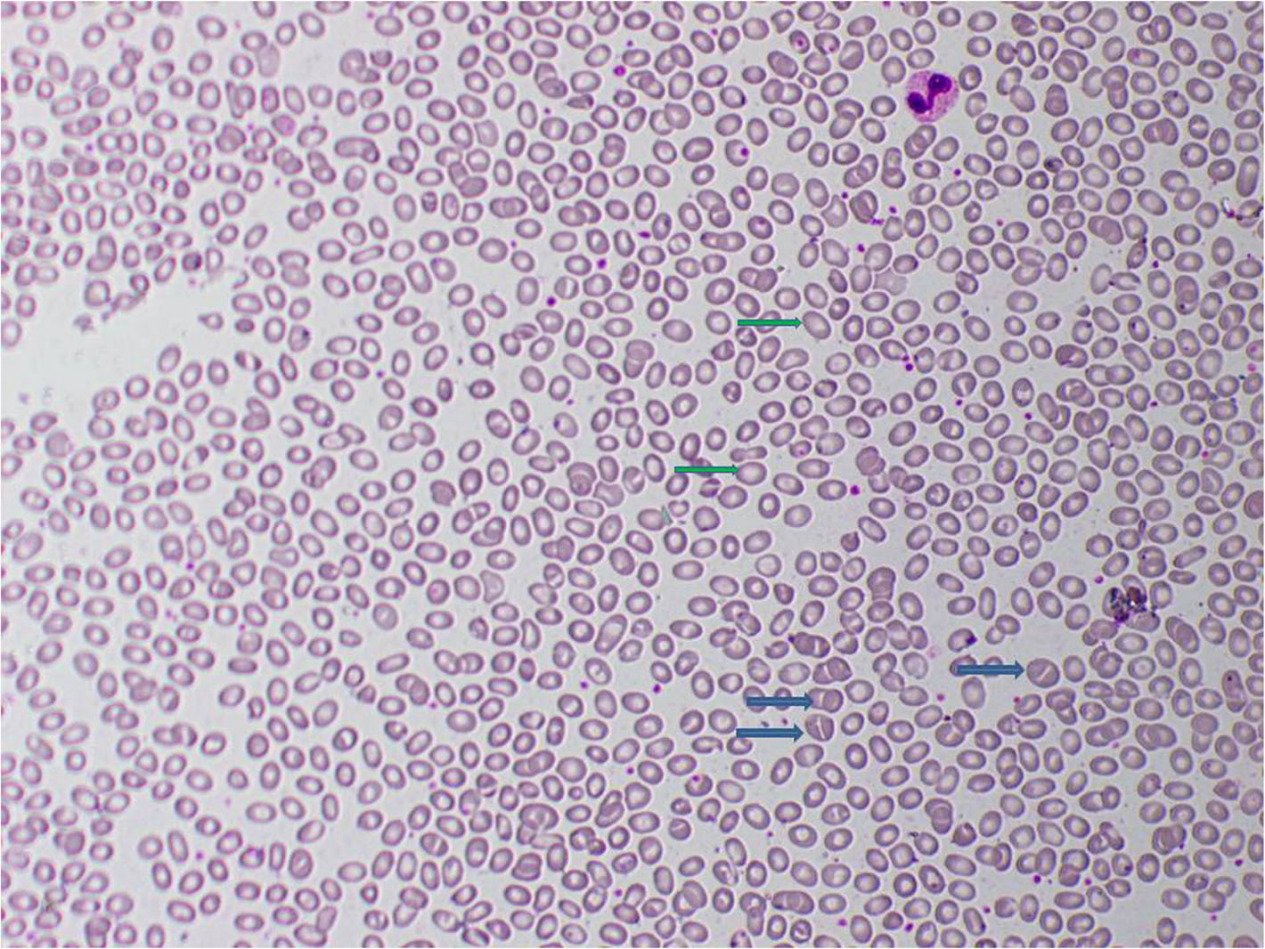
Blood picture of SAO. Stomatocytes (blue arrows) and macro ovalocyte (green arrows)

Further evaluation during this admission showed evidence of chronic kidney disease (CKD) and bilateral medullary nephrocalcinosis. Her blood gas analysis revealed a metabolic acidosis with alkaline urine. The presence of high anion gap with low delta ratio indicated mixed high and normal anion gap metabolic acidosis due to CKD and dRTA respectively (Table 1). She had low serum calcium, normal phosphate, high alkaline phosphate and high intact parathyroid hormone levels. Her x-rays showed reduced bone density, looser zones, and pathological fracture compatible with osteomalacia. In addition there was resorption of distal ends of long bones due to secondary hyperparathyroidism (Figs. 2, 3 & 4). Her hemoglobin was 9.3 g/dl with high mean corpuscular volume (MCV) of 106.6 fl. Her blood picture showed stomatocytic macro-ovalocytes compatible with SAO (Fig. 5).

### Patient 2

A previously healthy 59-year-old female from Kahatagasdigiliya, (Fig. 1) was admitted with gradual onset paralysis of lower limbs over 2 days. There was no family history of renal disease or similar illness among her eight siblings. She had flaccid paralysis of lower limbs with muscle power of grade 4 MRC, normal reflexes and normal sensory system. Examination of other systems was unremarkable. She was severely hypokalemic (serum potassium 2.3 mmol/L), and further investigations revealed normal anion gap metabolic acidosis high urine pH compatible with dRTA (Table 1). Her serum calcium was marginally low but there was no biochemical or radiological evidence of metabolic bone disease (MBD). Her haemoglobin was normal with elevated MCV of 103 fl. Her blood picture showed stomatocytes and ovalocytes compatible with SAO.

### Patient 3

**Fig. 6:**
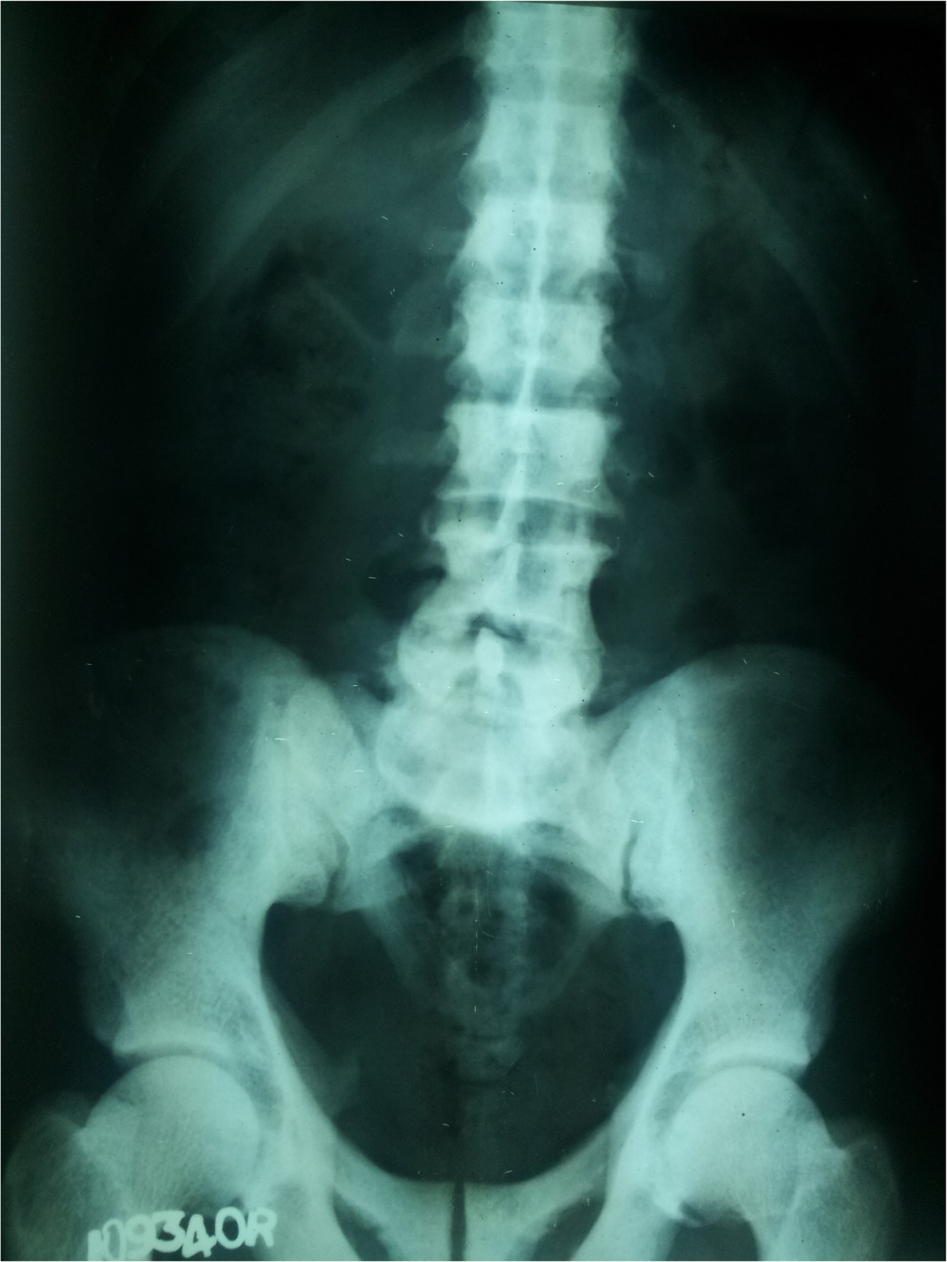
X ray lumbosacral spine showing diffuse osteosclerosis

A 62-year-old previously well male patient from Alayapaththuwa (Fig. 1) presented with gradual onset weakness of both upper limbs and lower limbs over days. His muscle power in lower limbs was MRC grade 3and upper limbs grade 4 with low serum potassium (2.1 mmol/L). Remainder of the physical examination was unremarkable. His serum creatinine was elevated and ultrasound scan of the kidneys showed evidence of CKD. He had compensated metabolic acidosis and despite that his urine was alkaline (Table 1). Presence of high anion gap and low delta ratio is probably due to the co-existing CKD and RTA. He had low serum calcium, elevated alkaline phosphate and elevated parathyroid hormone levels. His lumbosacral spinal X-rays were diffusely osteosclerotic. (Fig. 6). He was mildly anemic with haemoglobin value of 10.6 g/dl with elevated MCV (109 fl). His blood film was compatible with SAO.

### Patient 4

**Fig. 7:**
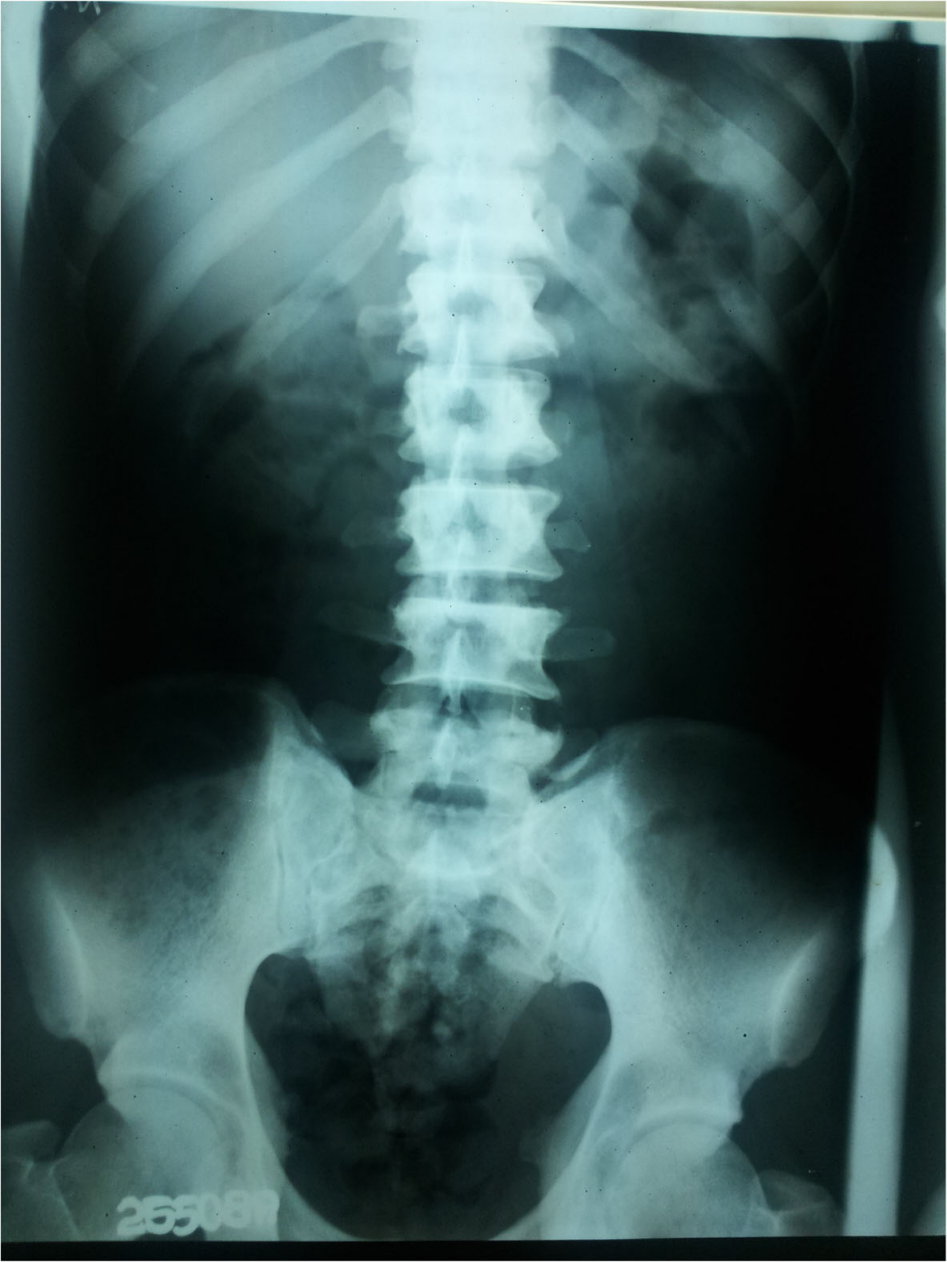
X ray lumbosacral spine showing diffuse osteosclerosis

A 32-year-old male from Thanthrimale (Fig. 1), was admitted with flaccid paralysis of lower limbs developed over 2 days. Within last 2 years, he had two prior admissions with paralysis of limbs attributed to significant hypokalemia, which improved after potassium replacement. He was on potassium chloride tablets. Present admission was following self-withdrawal of his medication. In addition he complained of lower back pain for 2 months. His father died of an undiagnosed renal disease. Neurological examination revealed muscle power grade 4 MRC in both lower limbs with intact reflexes and normal sensory examination. Examination of other systems was unremarkable. He had hypokalemia (serum potassium 2.5 mmol/L). Blood gas analysis showed compensated metabolic acidosis with high anion gap and a low delta ratio indicating mixed normal and high anion gap metabolic acidosis. Despite systemic acidosis his urine was alkaline which led to the diagnosis of renal tubular acidosis (Table 1). His ultrasound scan of kidneys showed evidence of chronic renal parenchymal disease. He had hypocalcaemia, normal phosphates, high alkaline phosphatase and normal parathyroid hormone levels. X-ray of lumbo-sacral spine and chest, revealed evidence of sclerotic metabolic bone disease (Fig. 7). He also had normal haemoglobin and raised MCV of 103.9 fl. His blood film revealed evidence of SAO.

### Patient 5

A 55-year old male from Medawachchiya (Fig. 1) was under investigation for periodic paralysis due to hypokalemia for 3 years. There was no family history or similar presentation among his ten siblings. He was admitted with muscle power of MRC grade 4 in upper and lower limbs. Examination of other systems was unremarkable. His serum potassium on admission was 2 mmol/L. Paralysis improved with intravenous potassium. Subsequent investigations revealed compensated hyperchloremic metabolic acidosis with normal anion gap. Despite systemic acidosis he had high urinary pH indicating renal tubular acidosis (Table 1) and his blood picture was compatible with SAO. Renal ultrasound scan revealed bilateral medullary nephrocalcinosis. He did not have biochemical or radiological evidence of metabolic bone disease. He was not anemic but had high MCV of 105 fl. His blood smear was compatible with SAO.

### Patient 6

A 50-year-old female from Padaviya (Fig. 1) with past history of periodic paralysis due to hypokalemia and hypothyroidism, presented with weakness of lower limbs and widespread myalgia. She was on thyroxin, but not on potassium tablets. There was no significant family history. She had generalized muscle weakness but her tendon reflexes, plantar responses and sensory examination were normal. Except for grade two multi nodular goiter remainder of the examination was unremarkable. Investigations revealed hypokalemia (serum potassium 2.4 mmol/L), compensated metabolic acidosis with normal anion gap and alkaline urine indicating RTA (Table 1). She had marginally low serum calcium, normal phosphate and high alkaline phosphatase suggestive of osteomalacia, but her x-rays were normal. Her haemoglobin was normal and blood picture was compatible with SAO.

### Management

All were advised on the need for long-term treatment to minimize nephrocalcinosis and MBD associated with dRTA. They were started on oral sodium bicarbonate1–2 meq/kg/day aiming at serum bicarbonate 22 to 24 meq/L. Patients who were intolerant to or showed inadequate response to sodium bicarbonate were given potassium citrate. All are being regularly followed up in outpatient medical clinic.

## Discussion and conclusion

All patients had hypokalemia and metabolic acidosis indicated by low serum bicarbonate (Table-1). Distal RTA (type-1) was confirmed by normal anion gap, hyperchloraemia, high urine pH and positive urine anion gap. Association with SAO could also help the diagnosis and suggest the hereditary nature of the dRTA. Patient 1 in 2016, and patient 3 and 4 had developed CKD and mixed metabolic acidosis (indicated by delta ratio) and all others had normal anion gap metabolic acidosis. Patient 1 & 5 had medullary nephrocalcinosis. All patients had SAO.

Two families in Sri Lanka with SAO were reported in 2004 without referring to the association with dRTA [6]. In the first family one grandparent was an immigrant from Malaysia and in the second family one parent was from Anuradhapura who had SAO. Another patient from Anuradhapura with dRTA, SAO and possible fluorosis was described in 2009 [7]. All six patients described above are from different regions of Anuradhapura district (Fig. 1). To their knowledge there were no Malayan descents in their families, and individual patients are not related to each other.

SAO has unique red cell morphology in the blood film with stomatocytes, ovalocytes, and macro-ovalocytes and found almost exclusively in South-East Asia [2]. The erythrocyte in SAO is exceptionally rigid, the abnormality is believed to have evolved because it offers protection against certain forms of malaria [8, 9]. Two out of six patients described were mildly anemic and both of them had CKD. There was no evidence of hemolysis in any of them as suggested by normal reticulocyte count (Table 1). All had elevated MCV possibly owing to macro ovalocytes.

Distal RTA is often associated with nephrocalcinosis, hypokalaemia and metabolic bone disease. Progression of nephrocalcinosis may lead to development of CKD [10]. If detected early in life, correction of the acidosis by alkali therapy could arrest progression of chronic kidney disease, and metabolic bone disease. Correction of serum potassium with Potassium Chloride salt is not appropriate without adequate metabolic control with alkali therapy. However, the pathophysiology of CKD is unclear and probably several mechanisms are involved including repeated pre renal acute kidney injuries, tubulo-interstitial damage due to nephrocalcinosis and persistent hypokalemia. Around 80% of patients will develop some degree of renal impairment although the progression to end stage kidney disease is delayed [10, 11].

The usual type of metabolic bone disease (MBD) in dRTA is osteomalacia. There are rare case reports of diffuse osteosclerosis [12] and MBD with hyperparathyroidism [13] secondary to dRTA. Our first patient had osteomalacia with secondary hyperparathyroidism due to chronic metabolic acidosis caused by dRTA. It was complicated with a pathological fracture. Third and fifth patients had biochemical evidence of osteomalacia and radiologically there was diffuse osteosclerosis. In addition, third patient had mild elevation of intact parathyroid hormone levels (Table 1).

Assessment of CKD in three out of six patients was challenging as recently there was an epidemic of CKD of toxic origin termed chronic interstitial nephritis in agricultural communities (CINAC) in the same area of Anuradhapura district [14]. Although there is hypokalaemia in both these patient groups CINAC patients have naturesis and hyponatraemia suggesting a proximal as opposed to distal tubular pathology seen in our patients. CINAC patients have small echogenic kidneys with decreased cortico-medullary ratio but medullary nephrocalcinosis is not usually seen as in dRTA.

Six adult patients, 3 males and 3 females age ranging from 32 yrs. to 62 yrs. with dRTA in association with SAO, suggesting hereditary dRTA, were evaluated here. Recurrent admissions with hypokalemia with paralysis presenting during adult life, presence of several types of MBD were few distinctive feature in this cohort. Inability to perform genetic testing due to unavailability of resources in Sri Lanka was a limitation in this study.

In conclusion, SAO and dRTA combination is less well described in Sri Lanka. A single case described earlier is also from Anuradhapura district of Sri Lanka. Mutations of *SLC4A1* gene may have evolved as SAO has protection against malaria and north central part of the country was repeatedly affected by malaria infection starting from 1029 AD. Six patients presenting to Anuradhapura hospital within 6 months indicate that the number would be more and probably under diagnosed. This case series highlight the importance of investigating patients presenting with hypokalemic paralysis and periodic paralysis as one cause would be dRTA. Early diagnosis and correction of acidosis are important in preventing complications. Delay in treatments lead to development of nephrocalcinosis, chronic kidney disease and metabolic bone disease.

## Data Availability

The datasets used during the current study are available from the corresponding author on reasonable request.

## Abbreviations

dRTA: Distal renal tubular acidosis
SAO: Southeast Asian ovalocytosis
MBD: Metabolic bone disease
CKD: Chronic kidney disease
CINAC: Chronic interstitial nephritis in agricultural communities
